# Curcumin inhibits HIV-1 by promoting Tat protein degradation

**DOI:** 10.1038/srep27539

**Published:** 2016-06-10

**Authors:** Amjad Ali, Akhil C. Banerjea

**Affiliations:** 1 Laboratory of Virology, National Institute of Immunology, New Delhi, India

## Abstract

HIV-1 Tat is an intrinsically unfolded protein playing a pivotal role in viral replication by associating with TAR region of viral LTR. Unfolded proteins are degraded by 20S proteasome in an ubiquitin independent manner. Curcumin is known to activate 20S proteasome and promotes the degradation of intrinsically unfolded p53 tumor suppressor protein. Since HIV-1 Tat protein is largerly unfolded, we hypothesized that Tat may also be targeted through this pathway. Curcumin treated Tat transfected HEK-293T cells showed a dose and time dependent degradation of Tat protein. Contrary to this HIV-1 Gag which is a properly folded protein, remained unaffected with curcumin. Semi-quantitative RT-PCR analysis showed that curcumin treatment did not affect Tat gene transcription. Curcumin increased the rate of Tat protein degradation as shown by cycloheximide (CHX) chase assay. Degradation of the Tat protein is accomplished through proteasomal pathway as proteasomal inhibitor MG132 blocked Tat degradation. Curcumin also decreased Tat mediated LTR promoter transactivation and inhibited virus production from HIV-1 infected cells. Taken together our study reveals a novel observation that curcumin causes potent degradation of Tat which may be one of the major mechanisms behind its anti HIV activity.

The majority of cellular protein degradation occurs through the two pathways, the lysosomal and the proteasomal pathway^1^,^2^. Proteasomal pathway is a specific and controlled process in which the substrate protein is tagged with ubiquitin and degraded by 26S proteasome complex^3^. Proteins are also degraded through the 20S proteasomes where the degradation is independent of the ubiquitination process^4^,^5^. Partially or completely unstructured proteins as well as oxidized proteins are degraded through this pathway^6^,^7^. The unstructured proteins are normally stabilized in the cell by associating with other proteins or protein complexes^8^. The unstructured proteins are also protected by NADH bound NAD(P)H:quinone oxidoreductase 1 (NQO1) protein present on the 20S proteasome^4^,^8^. Competitive inhibitors of NADH, like dicoumarol and curcumin release NADH from NQO1 rendering the 20S proteasome active for the degradation of unstructured proteins^9^,^10^,^11^. Many of the cellular proteins which are either partially or completely unfolded in their native condition, are degraded through this pathway including p53, p73, BimEL, PGC1 alpha etc^10^,^11^,^12^,^13^,^14^.

The pathogenic potential of HIV-1 is due to its rapid replication, spread and successful neutralization of host restriction factors which is mediated by its regulatory and accessory proteins^15^. HIV-1 Tat increases viral replication, by binding with TAR region in viral RNA and enhancing its transcription^16^. Tat is a small (86–101 amino acids) and intrinsically unfolded protein which helps Tat to interact with multiple cellular proteins influencing multiple cellular pathways^17^,^18^.

Curcumin has long been known to possess anti HIV activity due to its effect on the HIV-1 protease, integrase and LTR^19^,^20^,^21^,^22^. Curcumin is an inhibitor of protease, integrase and it also inhibits NF-κB pathway which is important for HIV-1 gene expression^20^,^21^,^22^. The p53 tumor suppressor protein that possesses intrinsically unfolded regions is also degraded through ubiquitin independent 20S proteasomal pathway by curcumin^4^,^11^. Given the fact that curcumin is an activator of ubiquitin independent protein degradation pathway and Tat is an intrinsically unfolded protein^18^, in this report we have investigated whether curcumin also degrades HIV-1 Tat. Our results show that curcumin increases the rate of Tat protein degradation and the degradation process is carried out by the proteasomal pathway. The effect of curcumin mediated Tat degradation is also reflected in its functions namely the HIV-1 LTR promoter transactivation and virion production. Furthermore, curcumin treatment of chronically infected HIV-1 cells also results in potent inhibition of virus production.

## Materials and Methods

### Plasmids and Chemicals

HIV-1 proviral clone, pNL4-3 was kindly gifted by Malcolm Martin through NIH AIDS Reagent Program^23^. Myc-Tat was derived from pNL4-3 and cloned in pCMV-Myc plasmid (Clontech, USA), to obtain Myc- tagged Tat expression construct as described previously^24^. The 6X-His-Ub was a kind gift from Dmitri Xirodimas Dundee University, UK^25^. The HA-Ubiquitin KO, having all lysines mutated to arginine was kindly provided by Ted Dawson^26^. The cell line HEK-293T was purchased from ATCC. The TZM-bl cells containing ß-galactosidase and luciferase gene downstream of HIV-1 LTR promoter was obtained from NIH AIDS Reagent Program^27^. The J1.1 cells are Jurkat E6.1 derived cells were also obtained from NIH AIDS Reagent Program^28^. The Codon optimized HIV-1 Gag expressing plasmid gag-opt was kindly provided by Beatrice H. Hahn^29^. The cell line HEK-293T and TZM-bl cells were maintained in DMEM (Hi-Media, India) supplemented with 10% fetal calf serum (Invitrogen, USA), 100 U/ml penicillin and 100 μg/ml streptomycin (Invitrogen, USA) at 37 °C with 5% CO_2_ in a humidified incubator. The cell line J1.1 was maintained in RPMI (Hi-Media, India) supplemented with 10% fetal calf serum and antibiotics. Transfection was carried out using Lipofectamine 2000 (Invitrogen, USA) reagent using the manufacturer’s protocol. MG132, chloroquine, polybrene and PYR-41^30^ were obtained from Sigma, USA. Curcumin (Merck, Germany) stock solution of 20 mM **was** prepared in DMSO, which was diluted in the complete DMEM medium before adding to the cells.

### Western blotting and cycloheximide chase assay

Cell lysate was made using RIPA Lysis buffer (1% NP-40, 20 mM TrisCl, pH 7.5, 150 mM NaCl, 1 mM Na_2_ EDTA, 1 mM EGTA, 1% Sodium deoxycholate, 1 mM Na_3_VO_4_), separated on SDS-PAGE and transferred on nitrocellulose membrane. Membrane was blocked with 5% skim milk solution in 1X PBS (Phosphate Buffered Saline; 137 mM NaCl, 2.7 mM KCl, 10 mM Na_2_HPO_4_, 1.8 mM KH_2_PO_4_), washed thrice with 1X PBS containing 0.1% Tween 20 (PBST). Thereafter incubated with primary antibody, washed with PBST and probed with horseradish peroxidase (HRP)-conjugated secondary antibody. The blots were developed using ECL (Enhanced Chemi-luminiscent) reagent.

### Antibodies

Anti-c-myc monoclonal antibody (Clontech, USA; 1:1,000), anti-GAPDH antibody (Cell Signaling Technology, USA; 1:10,000), anti-6X-His monoclonal antibody (Sigma; 1:2,000), anti-p24 monoclonal antibody (NIH AIDS Reagent Program, USA; 1:3,000), anti-PARP(Cell Signaling Technology, USA; 1:1000), anti-Rabbit IgG conjugated to HRP (Jackson Immunoresearch, USA; 1:10,000) and anti-Mouse IgG conjugated to HRP (Jackson Immunoresearch; 1:10,000) were used for the western blotting experiments.

### Luciferase Assay

TZM-bl cells were grown in a 24 well plate and transfected with 0.2 μg of Myc-Tat expressing plasmid. After 36 hrs of transfection, curcumin was added and incubated for 12 hrs. The lysate was prepared and luciferase activity was measured with equal amount of proteins using Promega Luciferase Assay kit (Promega, USA) in TECAN luminometer (TECAN, Austria). Untransfected cells were also treated in a similar way and luciferase activity was measured.

### HIV-1 infection in TZM-bl cells

To study the effect of curcumin on the replication of HIV-1, 1 μg of pNL4-3 was transfected in a 35 mm HEK-293T dish and incubated for 24 hrs. Subsequently fresh complete medium along with indicated amounts of curcumin was added and the cells were incubated for 12 hrs. The viral supernatant was mixed with 1 ml of incomplete DMEM medium containing 4 μg/ml of polybrene and added to TZM-bl cells for 4 hrs. Thereafter cells were washed twice with incomplete DMEM and replaced with fresh complete DMEM and further incubated for 24 hrs.

### Direct ELISA for p24 measurement in the viral supernatant

To measure the extent of released virions from HIV-1 infected cells, we developed a direct ELISA method for estimating p24 protein. The viral supernatant was applied on ELISA plate and incubated overnight followed by blocking it with 5% skim milk solution. Subsequently the p24 Gag antibody was added to the wells and incubated for 4–5 hrs followed by addition of horseradish peroxidase (HRP)-labeled anti-mouse secondary antibody. Finally the wells were washed and HRP substrate TMB (3,3′,5,5′-Tetramethylbenzidine) was added. The plate was incubated at 37 °C for 5–10 minutes for color development followed by the addition of stop solution (2 N H_2_SO_4_). The reading was taken using ELISA reader at 450 nm wavelength.

### Preparation of Tat supernatant and its application to HEK-293 T cells

HEK-293T cells (10 cm dish) were transfected with 20 μg pCMV-Myc-Tat plasmid for 24 hrs . The supernatant was collected and applied to a separate dish of HEK-293T cells and incubated for 4 hrs. Thereafter curcumin (diluted into fresh DMEM) was added and incubated for 6 hrs.

### Reverse Transcriptase-Polymerase Chain Reaction (RT-PCR)

HEK-293T cells were transfected with Myc-Tat for 36 hrs followed by curcumin treatment as described before, total RNA was isolated using Trizol reagent as described by the manufacturer (Invitrogen) and reverse transcribed to form complementary DNA (cDNA) using cDNA synthesis kit (Promega). RNA (1 μg) was mixed with random primers and incubated at 70 °C for 15 minutes and kept at 4 °C. Reverse transcription mix containing 1X reaction buffer, MgCl_2_, dNTPs, rRNasin and reverse transcriptase enzyme was added and incubated at 25 °C for 5 minutes, 42 °C for 1 hr, 70 °C for 15 minutes and kept at 4 °C. This cDNA was used for PCR amplification (15 cycles) of Tat gene using Tat Forward Primer: 5′ATGGAGCCAGTAGATCCTAGACTAGAG3′ & Tat Reverse Primer: 5′CGTCGCTGTCTCCGCTTCTTCCT3′. GAPDH was used as control with primers; Forward: 5′ACCACCATGGAGAAGGCTGG3′ and Reverse: 5′CTCAGTGTAGCCCAGGATGC3′.

### *In vivo* ubiquitination Assay

The *in-vivo* ubiquitination assay was performed to detect ubiquitinated proteins as described before^31^. HEK-293T cells were co-transfected with Myc-Tat and 6XHis-Ub (6X Histidine-ubiquitin) plasmids for 24 hrs, subsequently the cells were treated with curcumin or MG132 for the required time periods. Cells were lysed and ubiquitinated proteins were purified under denaturing conditions in guanidine hydrochloride buffer using Ni-NTA affinity chromatography. The purified products were resolved on 10–12% SDS-PAGE and immuno-blotted.

### Curcumin Treatment of Jurkat J1.1 cells

TNF-α stimulated J1.1 cells were treated with curcumin for 12 hrs as indicated and cell culture medium containing virions were collected. The virus containing medium was used to measure p24 level by ELISA an indicator of released virions from the cells. The virus producing Jurkat J1.1 cells were lysed and probed for p24 antigen by immunoblotting to measure virus replication in these cells.

## Results

### Curcumin induces Tat protein degradation

**Figure 1:**
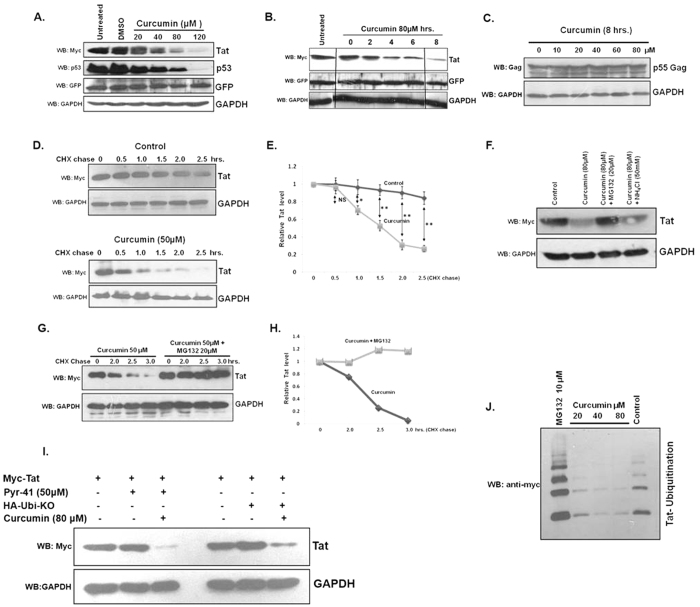
Curcumin decreased HIV-1 Tat protein. (**A**) HEK-293T cells were transfected with 1 μg of Myc-Tat expressing plasmid, and after 36 hrs treated with curcumin for 8 hrs, lysed and probed for Tat, p53 and GAPDH. pEGFP-N1 (50 ng) was also transfected as transfection control. The blot shown is a representative of three independent experiments.(**B**) Myc-Tat was transfected in HEK-293T cells and curcumin treatment was performed for increasing time period. The blot is a representative of three independent experiments. (**C**) Gag-Opt (1 μg) was transfected and curcumin treatment was performed followed by immmuno-blotting for Gag protein in HHEK-293T cells. (**D**) Myc-Tat transfected HEK-293T cells were treated with CHX alone or with curcumin for time periods as indicated and Tat protein level was measured by western blotting. (**E**) The mean value of Tat protein from three independent experiments was plotted with respect to treatment period. P value was calculated by a two-tailed t-test (*P < 0.05, **P < 0.01; NS, not significant). (**F**) The Myc-Tat transfected HEK-293T cells were treated with curcumin, along with proteasomal and lysosomal inhibitors MG132 and ammonium chloride for 8 hrs, subsequently Tat level was measured. (**G**) Myc-Tat transfected HEK-293T cells were treated with curcumin and CHX in the absence or presence of MG132 for different time periods followed by western blotting for Tat protein. (**H**) Densitometry of Tat bands was carried out by using image J and plotted with respect to treatment period. (**I**) HEK-293T cells were transfected with 1 μg of Myc-Tat (lanes 1–3) for 36 hrs followed by treatment with curcumin and Pyr-41 for 6 hrs subsequently the western blotting was done for Tat protein. HEK-293T cells were transfected with 1 μg of Myc-Tat and 2 μg of HA-Ub KO, 36 hrs of transfection curcumin treatment was done for 6 hrs and Tat protein was blotted. (**J**) HEK-293T cells were transfected with 1 μg of Myc-Tat and 2 μg of 6X-His Ubiquitin plasmid, after 36 hrs the cells were treated with increasing dose of curcumin or MG132. The ubiquitinated proteins were purified using Ni-NTA affinity chromatography and ubiquitinated Tat was blotted using anti-Myc antibody. The original uncropped blot images for (**A–D,F,G,I,J**) are shown in supplementary information as supplementary figure.

Earlier reports, including ours have described the unfolded nature of Tat protein and using Foldindex program we found Tat to be completely unfolded along its entire length^18^,^24^. The intrinsically unfolded proteins are degraded through ubiquitin independent 20S proteasomal pathway. Curcumin is reported to activate the pathway as it is a competitive inhibitor of NADH NQO1 interaction^11^. To investigate the effect of curcumin on Tat protein, Myc-Tat transfected HEK-293T cells were treated with curcumin, and Tat protein level was measured by western blotting. The level of Tat protein showed a decrease with curcumin treatment in a dose dependent manner (Fig. 1A). The p53 protein is known to be degraded by curcumin^11^, hence as a positive control, p53 protein level was measured which also showed similar decrease (Fig. 1A). For transfection control EGFP was transfected which was unaffected by curcumin treatment suggesting the specificity of Tat degradation. To investigate the effect of curcumin on Tat in a time dependent manner, HEK-293T cells were transfected with 1 μg Myc-Tat expressing plasmid followed by treatment with 80 μM curcumin from 0–8 hrs. Tat protein level was decreased with time in response to curcumin treatment (Fig. 1B). Unlike Tat, HIV-1 Gag is a properly folded protein as found by using Foldindex program. The effect of curcumin on Gag was investigated with curcumin treatment of HEK-293T cells that were transfected with Gag-Opt plasmid^29^. Immunoblotting of Gag showed that it remains largely stable with curcumin treatment as there is no significant decrease in the level of Gag protein (Fig. 1C). To study the effect of curcumin on the rate of Tat protein degradation, cycloheximide (CHX) chase assay was carried out of Myc-Tat transfected and curcumin treated HEK-293T cells. CHX treatment led to Tat protein degradation in a time-dependent manner, however the treatment of curcumin and CHX together results in rapid degradation of Tat supporting the fact that curcumin induces Tat degradation (Fig. 1D,E). To investigate the degradation pathway involved, Myc-Tat transfected HEK-293T cells were treated with curcumin alone, or curcumin along with proteasomal inhibitor MG132 or lysosomal inhibitor ammonium chloride. Tat protein levels were decreased after treatment with curcumin alone or with curcumin and ammonium chloride, however it was unaffected when the cells were treated with curcumin and MG132 suggesting the involvement of proteasomal pathway in the process (Fig. 1F). To further confirm the finding CHX chase assay was carried out of Myc-Tat transfected HEK29T cells treated with curcumin or curcumin + MG132. The degradation of Tat protein was inhibited completely when MG132 treatment was carried out confirming the involvement of proteasomal degradation of Tat in response to curcumin treatment (Fig. 1G,H). The ubiquitination of proteins can be inhibited by the treatment of ubiquitin activating enzyme E1 inhibitor Pyr-41^30^ or with the expression of dominant negative ubiquitin (HA-Ubiquitin KO) having all the lysine residues replaced with arginine^26^. To study whether the degradation of Tat by curcumin is dependent on ubiquitination of Tat, Myc-Tat transfected HEK-293T cells were treated with Pyr-41 and curcumin. Pyr-41 treatment was unable to stop the curcumin mediated Tat protein degradation suggesting for ubiquitin independent degradation (Fig. 1I). Curcumin treatment of HEK-293T cells that were earlier transfected with Myc-Tat + HA-Ubiquitin KO also resulted in the complete degradation of Tat protein (Fig. 1I). In order to study the effect of curcumin on Tat ubiquitination, HEK-293T cells were transfected with Myc-Tat and 6X-His Ubiquitin followed by curcumin treatment for 8 hrs. MG132 treatment was also carried out, which increased the extent of Tat ubiquitination. The ubiquitination of Tat got decreased in a dose dependent manner with curcumin treatment (Fig. 1J). Thus the results show that curcumin specifically promotes the degradation of Tat, which is a structurally unfolded protein, the properly folded Gag protein is unaffected by curcumin treatment. The degradation of Tat was found to be through proteasomal pathway independent of ubiquitination.

### Curcumin inhibits the functional activity of Tat

**Figure 2:**
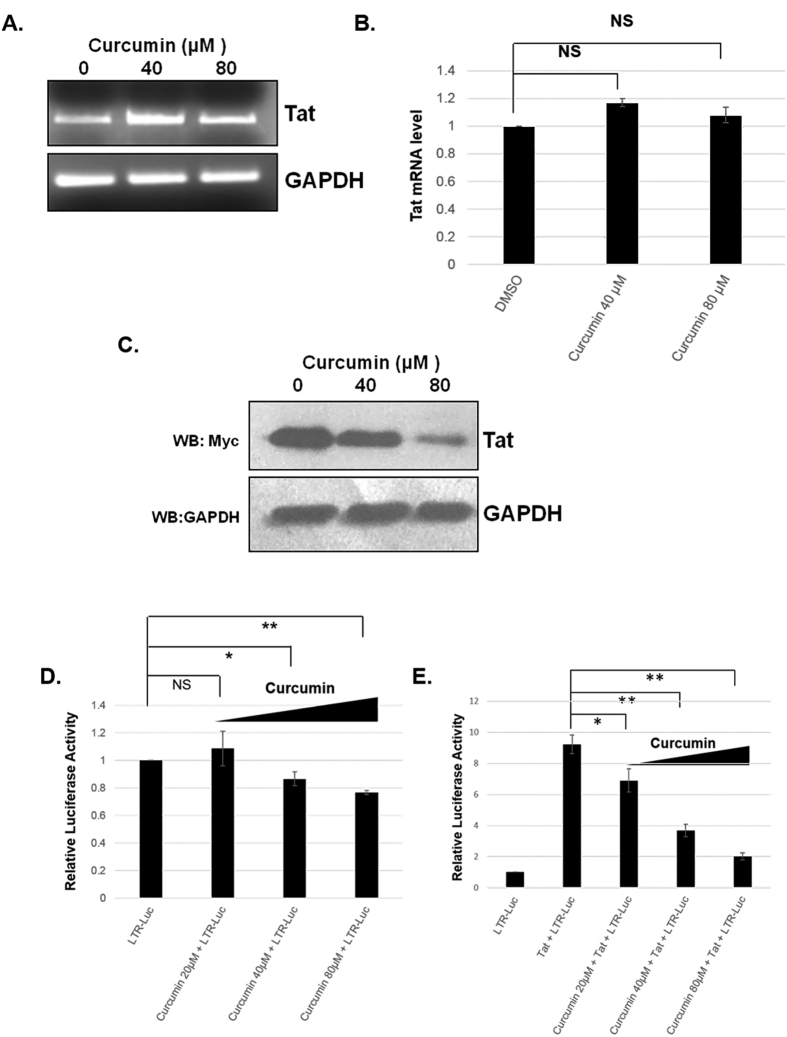
Curcumin does not modulate the level of Tat mRNA. (**A**) Myc-Tat transfected HEK-293T cells were treated with curcumin for 8 hrs and total RNA was isolated using TRIZOL reagent followed by RT-PCR using Tat and GAPDH primers. The gel image is a representative of three independent experiments. (**B**) Using ImageJ the band intensity of Tat and GAPDH was quantified and plotted as Tat/GAPDH. P value was calculated by a two-tailed t-test (*P < 0.05, **P < 0.01; NS, not significant). (**C**) Myc-Tat was transfected to HEK-293T cells, 24 hrs post transfection the cell culture medium was applied on fresh HEK-293T cells followed by treatment with curcumin for 6 hrs. Western blotting was done to detect the Tat protein. (**D**) In a 24 well plate format TZM-bl cells were transfected with 0.2 μg of Myc Tat, after 36 hrs treated with increasing dose of curcumin for 12 hrs and luciferase activity was measured and the mean of three independent experiments was plotted. P value was calculated by a two-tailed t-test (*P < 0.05, **P < 0.01; NS, not significant). (**E**) Similarly un-transfected TZM-bl cells were also treated with curcumin and luciferase activity was measured and the mean of three independent experiments was plotted. P value was calculated by a two-tailed t-test (*P < 0.05, **P < 0.01; NS, not significant).

To study the effect of curcumin on Tat mRNA, Myc-Tat transfected HEK-293T cells were treated with curcumin for 8 hrs followed by total RNA isolation and performing semi-quantitative RT-PCR assay. The results clearly showed that there is no change in the Tat cDNA level after curcumin treatment. As a control GAPDH mRNA was also amplified the level of which is also not changed (Fig. 2A,B). To completely rule out the role of curcumin on Tat transcription, Myc-Tat expressing plasmid was transfected in HEK-293T cells and the cell culture medium that contains Tat protein was applied to a fresh plate of HEK-293T cells followed by treatment with curcumin. Curcumin treatment reduced the Tat level suggesting the Tat protein degradation by curcumin occurs independent of its mRNA (Fig. 2C). Effect of curcumin on Tat functional activity was carried out using TZM-bl cell line harbouring an integrated HIV-1 LTR promoter upstream of luciferase gene^27^. The luciferase activity was measured in Myc-Tat transfected TZM-bl cells treated with curcumin for 12 hrs. Expression of Tat in TZM-bl cells resulted in an increase in luciferase activity by 9 folds which was reduced by 60% with 20 μM and 80% with 40 μM curcumin (Fig. 2D). Effect of curcumin on Tat independent HIV-1 LTR activation was also carried out by curcumin treatment of TZM-bl cells. There is a 20% decrease in LTR promoter activity at 40 μM and 30% decrease at 80 μM curcumin which is significantly less in comparison to Tat induced LTR activity (Fig. 2E). The role of curcumin on Tat gene transcription was ruled out by performing semi-quantitative RT-PCR as well as studying the effect of curcumin on Tat protein treated cells. The functional effect of curcumin on Tat was confirmed by performing LTR-luciferase assay in Tat transfected and curcumin treated cells.

### The HIV-1 virion production is inhibited with curcumin treatment

**Figure 3:**
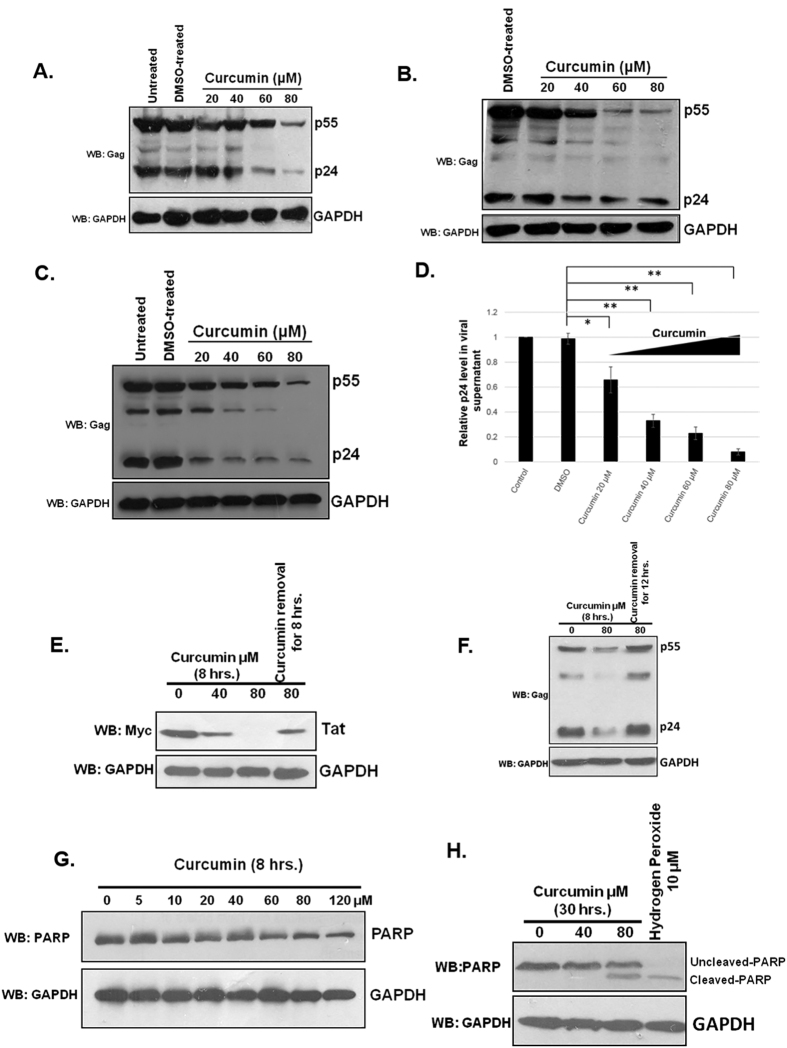
Curcumin treatment resulted in a reduced production of HIV-1 virions. (**A**) HEK-293T cells were transfected with 1 μg pNL4-3, after 36 hrs the medium was changed and curcumin was added from 20–80 μM for 12 hrs. The cells were lysed and probed for p24 Gag protein. Medium containing viral supernatant was used to infect TZM-bl cells. (**B**) TZM-bl cells were infected with viral supernatant obtained from previous experiment and the cells were further incubated for 24 hrs followed by lysis and measurement of p24 level. (**C**) J1.1 cells were stimulated with 20 ng/ml of TNF-α for 12 hrs subsequently the medium was replaced with fresh complete RPMI containing curcumin and TNF-α as indicated. The cells were lysed and blotted for p24 level whereas the cell culture medium containing virions were used to detect viral load by direct ELISA. (**D**) The virus containing medium from previous experiment was coated on 96 well ELISA plate and probed with p24 antibody, the average value of p24 from three independent ELISA experiments was plotted with respect to curcumin concentration. P value was calculated by a two-tailed t-test (*P < 0.05, **P < 0.01; NS, not significant). (**E**) Myc-Tat transfected HEK-293T cells were treated with curcumin for 8 hrs subsequently it was removed and fresh complete DMEM was added and incubated for 8 hrs followed by immunoblotting for Tat. (**F**) Curcumin treatment was carried out to pNL4-3 transfected HEK-293T cells followed by replacement with fresh DMEM medium and further incubation for 12 hrs. The p24 protein level was measured by western blotting. (**G**) HEK-293T cells were treated with indicated doses of curcumin for 8 hrs followed by western blotting for PARP. (**H**) Curcumin treatment was carried out for 30 hrs followed by western blotting to detect un-cleaved and cleaved PARP. As a control the cells were treated with hydrogen peroxide and blotted for PARP.

Curcumin is known to target HIV-1 protease, integrase and cellular NF-κB protein for the downregulation of HIV-1 replication^19^,^20^,^21^,^22^. However since our results showed its role in the degradation of Tat, its effect on the HIV-1 virion production was investigated. pNL4-3 was transfected in HEK-293T cells followed by curcumin treatment for 12 hrs. Cells were lysed and probed for p24 protein and the cell culture medium was saved and used for infecting TZM-bl cells. In the pNL4-3 transfected HEK-293T cells p24 levels decreased in a dose dependent manner with curcumin treatment (Fig. 3A). The p24 level of virus infected TZM-bl cells also decreased by 30% at 20 μM curcumin and reached upto 90% at 80 μM concentration (Fig. 3B). We further examined the effect of curcumin on the viral replication and virion production from chronically infected human T cell line J1.1. It is a Jurkat E6.1 derived human T lymphocyte, chronically infected with HIV-1 LAI strain. Under normal growth conditions there is limited viral replication and release from these cells, which increases tremendously upon stimulation with TNF-α^28^. To investigate the effect of curcumin on HIV-1 production and release, these cells were treated with TNF-α for 48 hours to induce HIV-1 expression. Subsequently the medium was replaced with fresh medium containing TNF-α and curcumin and incubated further for 12 hours. The viral replication was measured by p24 quantification through immuno-blotting. The viral supernatant was used to measure released virions from these cells using direct ELISA as described in methods. Curcumin treatment resulted in the dose dependent reduction of p24 level in the cell lysate of J1.1 cells (Fig. 3C), as well as in the supernatant (Fig. 3D). These results clearly confirm the inhibitory effect of curcumin on HIV-1 production from T cells. To rule out the possibility of Tat protein being degraded by curcumin was due to cell death of treated cells, Myc-Tat transfected HEK-293T cells were treated with curcumin for 8 hrs, subsequently the media was replaced with complete fresh DMEM and further incubated the cells for 8 hrs. As shown in Fig. 3E, treatment of 80 μM curcumin led to complete degradation of Tat, however removal of it for 8 hrs resulted in the re-appearance of Tat protein. Similarly when pNL4-3 transfected HEK-293T cells were treated with 80 μM curcumin for 8 hrs there was ~70% decrease in p24 level, whereas removal of curcumin for 12 hrs resulted in a complete rebound of virus replication as measured by p24 level, suggesting that curcumin treatment did not damage the protein synthesis machinery (Fig. 3F). To measure apoptosis in curcumin treated cells, HEK-293T cells were treated with increasing dose of curcumin for 8 hrs followed by measurement of cleavage of Poly (ADP-Ribose) Polymerase (PARP)^32^. There was no cleavage of PARP even with 120 uM curcumin treatment (Fig. 3G). However, when treatment was extended for 30 hrs cleavage in PARP protein was observed with 80 μM curcumin (Fig. 3H). As a positive control of apoptosis the cells were treated with hydrogen peroxide that also led to PARP cleavage^33^. The reduction of Gag level both in pNL4-3 transfected HEK-293T cells and chronically infected J1.1 cells clearly showed the effect of Tat degradation on HIV-1 virion production. The reversibility of the effect of curcumin on both Tat level as well Gag level in pNL4-3 transfected cells confirms that the effect of curcumin on Tat is not due to cell death which was further confirmed by measuring PARP cleavage in curcumin treated cells.

## Discussion

HIV-1 Tat is a structurally unfolded protein and gains its structure when associates with its partner cellular proteins^18^,^34^. In the cellular environment Tat protein must be present both in free form and in association with its partner proteins. The unassociated free Tat protein is unstructured and prone to degradation through ubiquitin independent 20S proteasomal pathway. NQO1 inhibits the degradation of Tat protein whereas dicoumarol which is an inhibitor of NQO1 enzymatic activity promotes its degradation^24^. NAD(P)H bound NQO1 protects many intrinsically unstructured proteins from degradation through 20S proteasomal pathway^9^,^10^,^11^,^12^. Dicoumarol and curcumin inhibit the NQO1 enzymatic activity and also displace NAD(P)H from NQO1 promoting the degradation of various proteins including p53^11^. Curcumin promotes the degradation of various proteins including cyclin D1, p185ErbB2, c-Jun, C/EBPα,, C/EBPβ and PGC1α, however the mechanism of degradation has not been worked out^11^,^35^. Besides it is clearly established that p53 is degraded through ubiquitin independent mechanism^11^. Curcumin also inhibits the proteasomal enzymes as well as deubiquitinating enzymes hence promote the accumulation of ubiquitinated proteins in general^36^. Thus general inhibition of 20S proteasomal pathway fails to inhibit the degradation of proteins many of which have intrinsically unstructured regions. These reports thus suggest that curcumin might be working separately as an inhibitor of proteasomal enzymes that leads to accumulation of ubiquitinated proteins, as well as inhibitor of the NAD(P)H-NQO1 interaction that leads to the degradation of unstructured proteins like p53, cyclin D1, p185ErbB2, c-Jun, C/EBPα,, C/EBPβ and HIV-1 Tat.

Our studies have clearly shown that curcumin promotes the degradation of Tat protein but leaving its mRNA level unaffected. The CHX chase assay and inhibition of degradation by MG132 clearly suggests the degradation of Tat protein is independent of the mRNA transcription which was further validated by demonstrating that Tat protein supernatant treated cells also showed degradation of Tat by curcumin. The treatment of Pyr-41 or expression of HA-Ubiquitin KO and subsequent treatment with curcumin clearly suggested that the degradation is independent of ubiquitination process. Tat protein gets ubiquitinated with wild type ubiquitin however the ubiquitinated fraction of Tat protein also gets reduced in response to curcumin treatment as like the total Tat protein. Curcumin also inhibits the functional activity of Tat namely HIV-1 LTR promoter transactivation. Tat independent LTR transactivation is also inhibited by curcumin albeit at a low rate since curcumin is an inhibitor of NF-κB transcription activity^21^. Curcumin treatment resulted in a decrease of p24 protein level both by western blotting and ELISA in both pNL4-3 transfected cells and chronically infected J1.1 cells showed the adverse functional effect of Tat degradation. The decrease in Tat level as well as p24 protein level is reversible as removal of curcumin led to reappearance of Tat protein as well as p24 protein. Thus the degradation of Tat is not due to the death of treated cells as there is no PARP cleavage after 8 hrs of treatment and PARP was cleaved when treatment was performed for extended time periods (30 hrs.). Curcumin has already been reported to inhibit the HIV-1 replication through a variety of mechanisms namely by the inhibition of its protease activity or by inhibiting the NF-κB mediated LTR transactivation^19^,^20^,^21^,^22^. Using multiple experimental approaches we have shown that curcumin rapidly induces specific degradation of Tat and this may explain why curcumin is a potent inhibitor of HIV-1 replication. In summary, our study establishes that curcumin mediated rapid degradation of HIV-1 Tat protein must be the major mechanism contributing to its potent anti-HIV-1 activity.

## Additional Information

**How to cite this article**: Ali, A. and Banerjea, A. C. Curcumin inhibits HIV-1 by promoting Tat protein degradation. *Sci. Rep.* **6**, 27539; doi: 10.1038/srep27539 (2016).

## Supplementary Material

Supplementary Information
