# Supplementary material for: Curcumin inhibits HIV-1 by promoting Tat protein degradation
Source: Sci Rep. 2016 Jun 10;6:27539. doi: 10.1038/srep27539 (PMC4901322; doi:10.1038/srep27539)
Supplement: Supplementary Information [file srep27539-s1.pdf]

Supplementary information

Curcumin inhibits HIV-1 by promoting Tat protein degradation.

Amjad Ali, Akhil C Banerjea*

Institutional Affiliations: Laboratory of Virology, National Institute of Immunology, New Delhi, India. Email: amjad139@gmail.com , akhil@nii.res.in.

***Correspondence author:** Dr. Akhil C. Banerjea, & **Amjad Ali,** Virology Lab, National Institute of Immunology, Aruna Asaf Ali Marg, New Delhi-110067, India; Tel: +91-011-26703616; Mobile: +91-9818717833; Fax: +91-011-26742125; Email: akhil@nii.res.in, akhil@nii.ac.in, amjad139@gmail.com

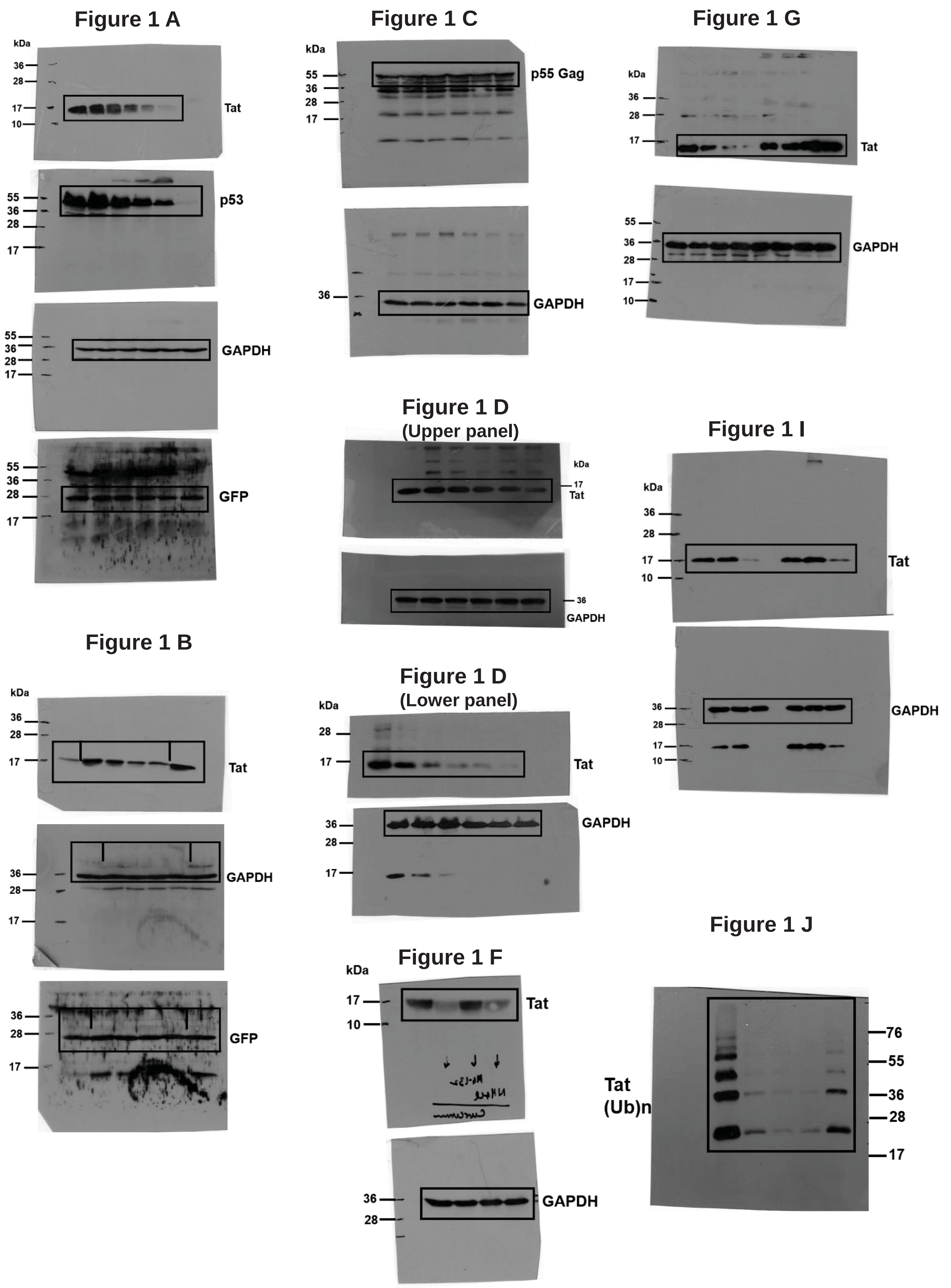

Supplementary Figure 1 : Uncropped images of western blots with molecular size marker (kDa).
